# Elementary Life

**Published:** 1887-02

**Authors:** Wm. A. Knowles


### Elementary Life.

BY WM. A. KNOWLES, M.D., D.D.S.

(Read before the California State Dental Association.)

Herbert Spencer says that "life is the continuous adjustment of internal relations to external relations." Professor White says "it is the continuous reactions of organized matter in response to incident forces." It has also been defined as "the manifestation of spiritual existence in the material form." Webster defines life as (1) "that state of an animal or plant in which its organs are capable of performing their functions; animate existence; vitality; also, the time during which this state continues, either in general or in an individual instance; as the life of a tree or a horse; (2) the time during which a human soul and body are united; the period between birth and death; the present state of existence; sometimes, the perpetual existence of the soul in the present and future state."

Where is the seat of life? We find life in bones, muscles, skin, nerves, and all other portions of the body; we find it in plants and in seeds. Life may apparently be suspended but still remain. It may lie dormant in seeds for thousands of years. Frogs and reptiles may become imbedded in stone formations and retain life for ages. A perfect definition of life, applicable to all cases, is difficult, if not impossible. In the human body the tripod of life consists of the brain, heart, and lungs, without any one of which life could not be possible. The brain governs and directs all actions, and the heart and lungs control the circulation. In the circulation, then, lies the secret of life.

The circulating fluid, the blood, consists of the liquor sanguinis and the red and white corpuscles. The liquor sanguinis is a fluid which bears nutrient material to all portions of the body to aid in the growth and repair of all tissues, and it also acts as a medium for the transportation of all broken-down tissues which are eliminated by the excretory organs in the form of effete matter.

The red corpuscles act as carriers of oxygen to the several tissues, and by union of the oxygen with carbon, form carbonic acid gas which is then returned to the lungs and passed out from the system. Life exists in these corpuscles, but it is life for one certain purpose.

The remaining constituent of the blood is the white corpuscle in which, then, must lie the secret of life.

These white corpuscles are small bodies somewhat larger than red corpuscles and are what are termed bioplasts. They normally exist in small quantities in the blood, but when an injury occurs to any part of the system, they begin to accumulate in larger numbers. They get to such situations by passing through the coats of the blood vessels. They are capable of being converted into any tissue of the body. They have the power of locomotion, moving about from place to place; they have the power of reproduction, multiplying and producing others of their kind, and have also the power of assimilation, or the absorption of other tissues for the purpose of aiding their own growth. These little cells, then, having these powers, contain all that comprises what we term " life."

If we examine any form of animal or plant life, we shall find these same life cells in modified forms, but it is from these cells that the elementary life is evolved. They build up and break down tissues, form bone, muscle, cartilage, sinew, skin, teeth, hair, nails, and all other varieties of tissue, keep them in repair, and when they no longer perform the office for which they were intended, they undo the work which they formerly created, and component portions are then carried away and cast out from the system.

In the animal system these bioplasts have received various names according to the positions they occupy. In the blood they are the white corpuscles; in bone and cementum they are termed osteoblasts; in dentine, odontoblasts; in enamel, they have been called ameloblasts (W. H. Eames), and in general all are classed under the term bioplasts. When the office of these bioplasts is to remove bone or dentine they have been termed osteo or odonto clasts (Wedl).

In the formation of teeth, which most concerns us, these little life cells form a layer upon the periphery of a soft and highly vascularized body. The blood vessels terminate in loops just under this layer of cells, and the liquor sanguinis by osmosis reaches these cells, giving them materials with which to work. These cells absorb the nutrient fluid, and in the innermost recesses of their laboratories this food or pabulum is converted into formed material and deposited upon the periphery of these cells. Layers after layers of these calcific deposits are formed until the whole cell may become obliterated, and by uniting with neighboring cells a finished tissue may be formed; or the deposit of calcium may not totally obliterate the central portion and a small life-center may remain to nourish and repair the tissues. Such cells always become impregnated with calcium in a direction from the periphery towards the center, and as each layer successively calcifies, others continually form behind them. They throw out processes through which the nutrient and formed material can be conveyed to other points, and the circulation of this fluid may keep open the small canals which are permanently found in the completed tissues. Small fibres of living matter remain between and around these calcific deposits and form a mesh of living matter binding all portions together. This process goes on until the tooth has assumed its perfect shape, and the odontoblasts then form a layer covering the pulp called the *membrana eboris.* This is for the repair of the tissues, to circulate the nutrient fluid in the tubuli, and to protect the teeth against mechanical abrasion incident to the natural wear on the teeth. In the temporary teeth these life cells form the active portion of the absorbent organ which removes the teeth of the first dentition to make way for a more useful and permanent denture.

Nature, in giving to man his denture, never intended that the teeth should become affected by caries. She recognized that they would be liable to suffer from mechanical abrasion and she provided against this very contingency.

The formation of secondary dentine, the zone of calcification keeps pace with the normal mechanical abrasion, and keeps the thickness of dentine between the irritated surface and the pulp sufficient to make it a non-conductor. This, in a permanent tooth, is the main object of the odontoblast, and is also the reason for the canaliculi remaining open, and for the fluid circulating in them, for it is the filling up of these tubuli which causes the condensation of structure.

Incidentally, we find the same reparative process occurring during the progress of caries, but the dentine cannot be repaired so rapidly as to protect the nerve from ultimate exposure. Nature has guarded against pulp irritation, but not from this direction.

Under the microscape both red and white corpuscles, as seen in the web of the frog, travel along the blood channels much as pedestrians are observed traveling along a street; some hurry along, others lag behind; some pause, some return, and some straggle off sideways from the road into adjacent tissues. Some intelligence guides and directs them. When any injury occurs they immediately congregate at that point, taking the shortest way to get there.

At other times they do not begin their work until a certain age or condition is reached, and they remain dormant until that time. Some are placed in certain positions to act in case of certain accidents. Thus a provision is made for new claws being formed in crabs, and in the higher orders of animals, in the splint which is formed about broken bones. These cells would remain quiescent for life should they never be called into action; and then, each one forms its appropriate tissue. The same bioplast forms skin, hair, nails, horns, teeth, hoofs, and feathers, and rarely misplaces one where the other tissues were intended to be developed.

Something governs these cells, but they are the workers, the builders, and the destroyers, and in them is to be found the secret of life. These may have no cell walls and perhaps no demonstrable nuclei, as neither are essential to the bioplast.

The constituent elements of bioplasts are carbon, hydrogen, oxygen, and nitrogen, and the composition is almost chemically dentical with albumen. When a bioplast is destroyed the resulting compounds are water, albumen, fat, and sometimes fibrin and salt.

In these elementary cells is not only stored up the powers of locomotion, assimilation, and reproduction, but there also seems to be an intelligence which guides them. Thus several cells which cannot be distinguished, one from another, will produce entirely different formations.

Through certain of these cells parental likenesses and peculiarities are transmitted, as is also disease from parent to child.

By these cells every different variety of plant and animal life is maintained.

In the same organization we see an intelligence guiding these cells. When an organ or set of organs becomes incapacitated others will assist to perform the work; thus, the kidneys and skin often assist each other. The intelligence guides cells in regard to the time when they shall be called into action.

When the amputation of an extremity occurs in utero in the human species, an effort is made to repair it and a rudimentary and diminutive substitute is supplied. The higher the type of organization, the less perfect the power of reproduction of lost parts, but an effort, at least, is made in the higher orders.

We cannot follow the secret of life to its very source, but it is certain that such secret is held by this elementary tissue, the bioplast.

